# Differential effects of real versus hypothetical monetary reward magnitude on risk-taking behavior and brain activity

**DOI:** 10.1038/s41598-018-21820-0

**Published:** 2018-02-27

**Authors:** Sihua Xu, Yu Pan, Zhe Qu, Zhuo Fang, Zijing Yang, Fan Yang, Fenghua Wang, Hengyi Rao

**Affiliations:** 1 Laboratory of Applied Brain and Cognitive Sciences, School of Business and Management, Shanghai International Studies University, Shanghai, China; 2 Department of Psychology, Sun Yat-sen University, Guangzhou, China; 3 Department of Psychology, Zhejiang University, Hangzhou, China; 4 Center for Functional Neuroimaging, Department of Neurology, Perelman School of Medicine, University of Pennsylvania, Philadelphia, PA USA

## Abstract

Human decisions are more easily affected by a larger amount of money than a smaller one. Although numerous studies have used hypothetical money as incentives to motivate human behavior, the validity of hypothetical versus real monetary rewards remains controversial. In the present study, we used event-related potential (ERP) with the balloon analogue risk task to investigate how magnitudes of real and hypothetical monetary rewards modulate risk-taking behavior and feedback-related negativity (FRN). Behavioral data showed that participants were more risk averse after negative feedback with increased magnitude of real monetary rewards, while no behavior differences were observed between large and small hypothetical monetary rewards. Similarly, ERP data showed a larger FRN in response to negative feedback during risk taking with large compared to small real monetary rewards, while no FRN differences were observed between large and small hypothetical monetary rewards. Moreover, FRN amplitude differences correlated with risk-taking behavior changes from small to large real monetary rewards, while such correlation was not observed for hypothetical monetary rewards. These findings suggest that the magnitudes of real and hypothetical monetary rewards have differential effects on risk-taking behavior and brain activity. Real and hypothetical money incentives may have different validity for modulating human decisions.

## Introduction

Human decisions are motivated by various kinds of rewards, including money as a secondary but strong incentive for regulating human behavior. Since money is universally valued, scalable, and can be used as both an incentive and a punishment, it is widely used for decision research both in the real world and in the laboratory^1–3^. Currently, numerous decision researches used hypothetical rather than real monetary rewards^4–6^, for the reason that real money incentives may be too costly and may have some ethical issues, especially for studies involving a large amount of monetary rewards. However, it remains controversial whether hypothetical and real monetary rewards modulate decision behavior and brain activity in the same way.

Although earlier studies found no differences between hypothetical and real rewards among different task paradigms^7–13^, accumulating evidences from more recent studies suggested that hypothetical and real monetary rewards may have differential influences on human decision behavior and brain activity^14–17^, particularly when the reward scale or magnitude is large^18,19^. For example, previous studies have shown that when participants were receiving real rewards during the discounting tasks, their choices were more self-controlled and less impulsive^18^. Significant behavior differences between real and hypothetical rewards were also observed in economic games including the sharing game, the ultimatum game, and the dictator game^15^. Using a Balloon Analogue Risk Task (BART) paradigm, our previous study found that individuals became more risk averse after negative feedback (i.e., money loss) with real compared to hypothetical monetary rewards^19^.

A few neuroimaging studies using functional magnetic resonance imaging (fMRI) and Electroencephalography (EEG) also suggested differential brain responses to real versus hypothetical rewards. For example, an fMRI study found stronger brain activation in the orbitofrontal cortex and ventral striatum when subjects made real purchases compared to hypothetical purchases^16^. A recent event-related potential (ERP) study demonstrated larger feedback-related negativity (FRN) in response to money loss during the BART risky decision making with real rewards compared to hypothetical rewards, which may reflect greater prediction error or regret emotion after real monetary losses^17^.

Apart from reward valence, reward magnitude (i.e., scale of payoff) also plays an important role in motivating human behavior. In general, both animals and humans prefer large rewards to small rewards^20–24^. For instance, participants would usually select more cards from disadvantageous decks with larger rewards when the reward magnitude was increased in the Iowa gambling task^23^. However, a few studies suggested that individuals may display different risk-taking and decision-making behavior changes to the increased magnitude of hypothetical versus real monetary rewards^19,25^. Specifically, participants became more risk averse when the scale or magnitude of real rewards increased, while no behavioral changes were found when the scale or magnitude of hypothetical rewards increased, suggesting an interaction between reward authenticity and magnitude on risk-taking behavior^19^. However, the neural bases for the potential differential effects of real versus hypothetical monetary reward magnitudes on risky decision-making behavior remain unclear.

**Figure 1:**
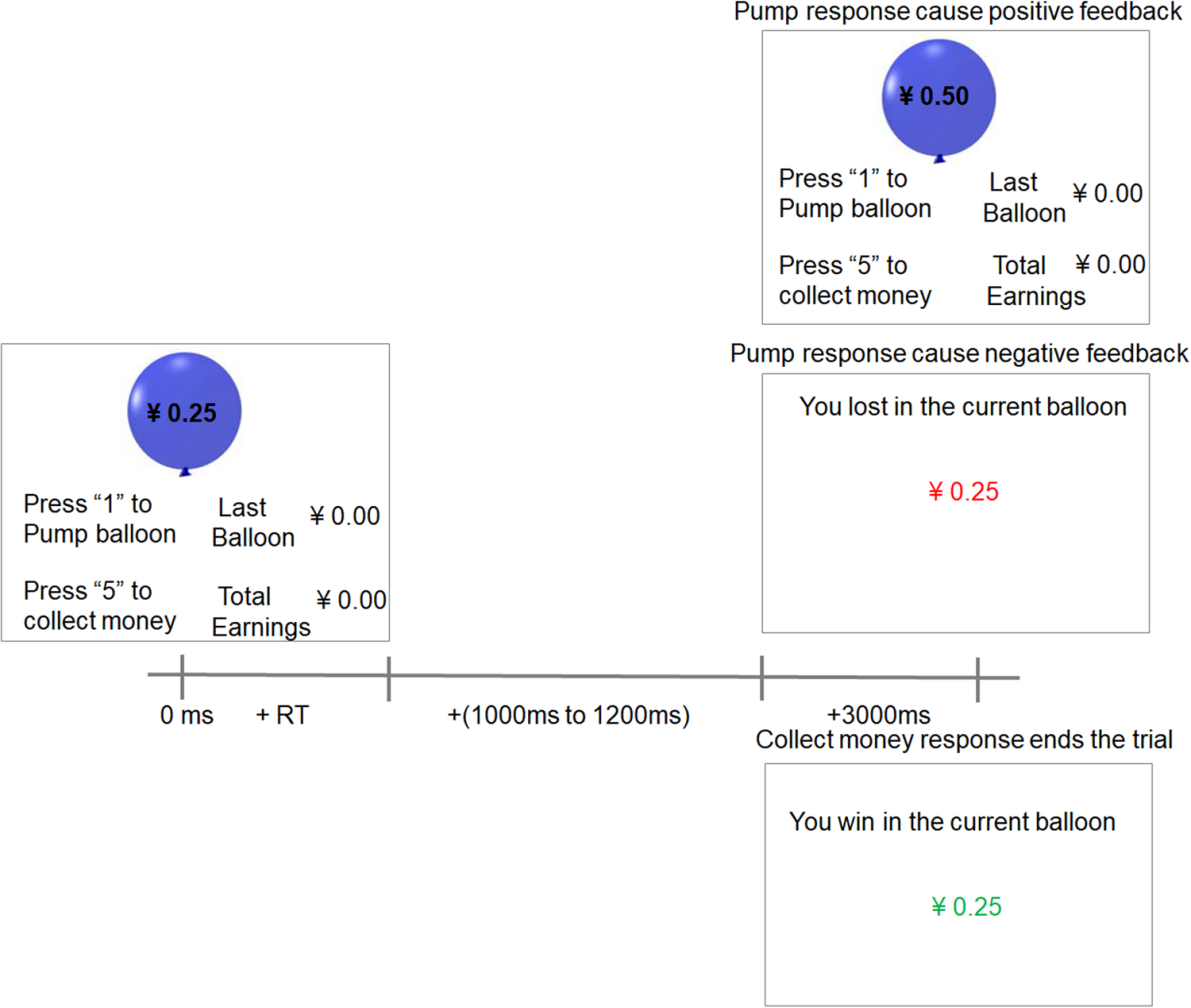
Schematic diagram for the BART. During the task, subjects were repeatedly given two options: press a button to continue inflating the balloon (the reward for each balloon increased with each inflation pump) or press another button to discontinue inflation. If subjects chose to stop inflation, they won the reward in the amount that the balloon was worth at the time they stopped inflation and the reward was added to their cumulative earnings. If subjects chose to continue inflation and the balloon exploded, they lost the reward for the current balloon, which was subtracted from the cumulative earnings as a penalty. Feedback stimuli were presented after a random delay (1–1.2 s) following subjects’ response.

In the present study, we used event-related potential (ERP) and measured brain responses to risk taking and decision making during the BART with large and small hypothetical or real monetary rewards, in order to examine the effects of real and hypothetical reward magnitude on risk taking in the brain. The BART is an ecologically validated cognitive task for the assessment of risk taking propensity and behavior^26–29^, in which participants are repeatedly given the option to continue or discontinue inflating a virtual balloon that could either grow larger or explode (see Fig. 1). Risk in the BART is typically defined as the probability of explosion for each balloon. The average number of inflation pumps participants make for the balloons provides an objective assessment of risk taking propensity^26–29^. We are specifically interested in the FRN component in response to negative feedback during the BART, because the FRN is a well-validated and widely-studied ERP component in the risk-taking and decision-making literature^3,17,30–34^. Previous studies have shown that the FRN is sensitive to outcome valence and magnitude and may reflect the evaluation of motivational and emotional consequences of decision outcomes^35–37^. We hypothesize that the magnitude of real monetary reward would show stronger influence than the magnitude of hypothetical monetary reward on the FRN after negative feedback (money loss) during the BART.

## Results

### Behavior Results

**Figure 2:**
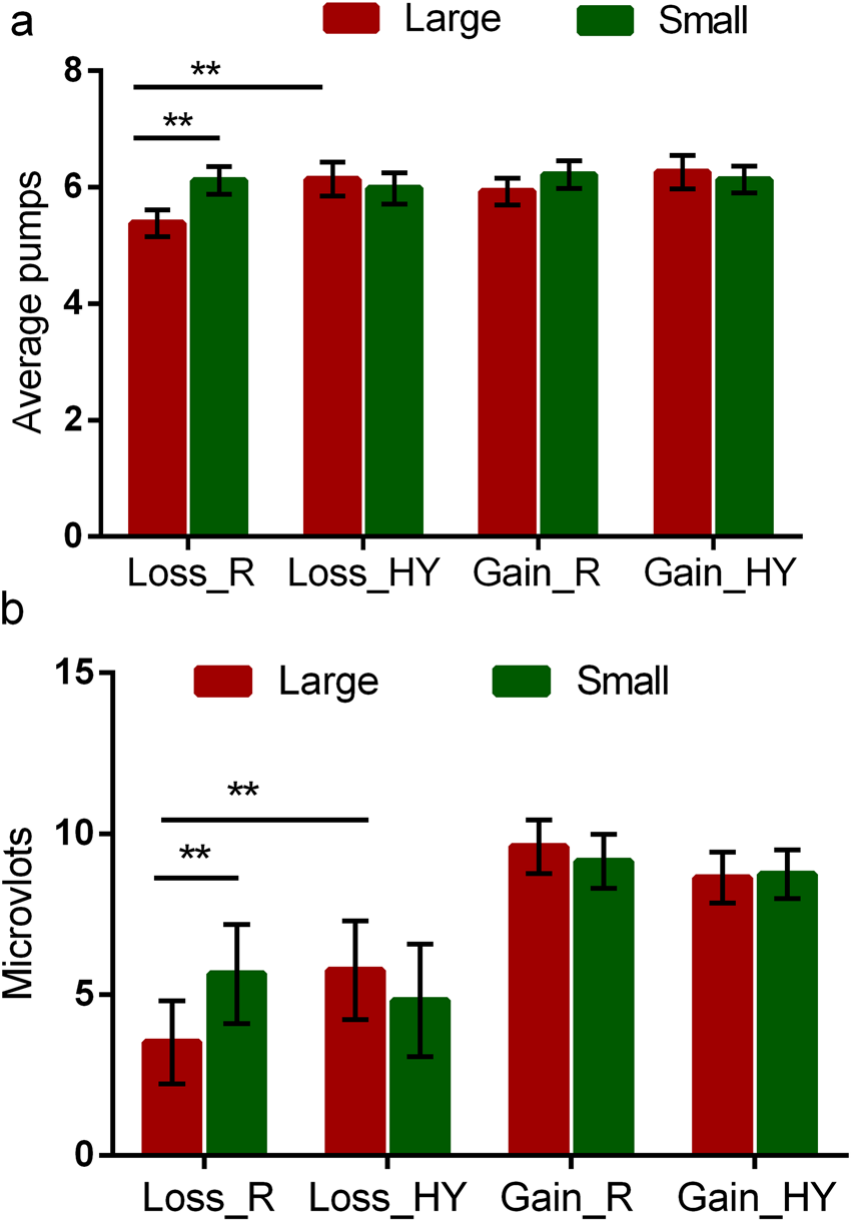
Behavior and ERP results from the BART with real and hypothetical monetary rewards. Average pumps following loss and win (gain) feedback for results of different scales (**a**), and the mean FRN amplitudes at Cz for four kinds of results in the 210–270 ms time window post-onset of feedback (**b**). Error bars represent standard error (SE). Loss_R, loss with real money; Loss_HY, loss with hypothetical money; Gain_R, gain with real money; Gain_HY, gain with hypothetical money. **Significant difference of *p* < 0.01.

A 2 (reward condition: hypothetical vs. real) × 2 (reward magnitude: small vs. large) × 2 (feedback: positive vs. negative) repeated measures ANOVA analysis was conducted on the average number of inflation pumps participants made for the balloons (Fig. 2a). This analysis revealed a significant three-way interaction among reward condition, reward magnitude, and feedback (*F* (1, 19) = 4.70, *p* = 0.043, *ω*^2^ = 0.158), and a significant main effect of feedback (*F* (1, 19) = 5.39, *p* = 0.031, *ω*^2^ = 0.179), with participants making less number of inflation pumps for the balloons following negative (loss) feedback than positive (win) feedback. We then separately analyzed the average number of inflation pumps participants made for the balloons following positive and negative feedbacks using 2 (reward condition: hypothetical vs. real) × 2 (reward magnitude: small vs. large) ANOVA, respectively. For the balloons following positive feedback, there were no effects of reward condition (*F* (1, 19) <1, *p* = 0.35) or reward magnitude (*F* (1, 19) <1, *p* = 0.75), and no interaction between reward condition and reward magnitude (*F* (1, 19) <1, *p* = 0.43). In contrast, for the balloons following negative feedback, there was a significant main effect of reward magnitude (*F* (1, 19) = 4.38, *p* = 0.047, *ω*^2^ = 0.144) and a significant interaction between reward condition and reward magnitude (*F* (1, 19) = 4.70, *p* = 0.043, *ω*^2^ = 0.156), but there was no main effect of reward condition (*F* (1, 19) = 2.10, *p* = 0.16, *ω*^2^ = 0.053). Further post-hoc comparisons indicated that the average number of pumps participants made for the balloons following negative feedback with large real monetary reward was significantly less than that with small real monetary reward (5.38 ± 0.23 vs. 6.12 ± 0.31, *t*(19) = −2.85, *p* = 0.01, *ω*^2^ = 0.091) and less than that with large hypothetical monetary reward (5.38 ± 0.23 vs. 6.14 ± 0.28, *t*(19) = −3.02, *p* = 0.007, *ω*^2^ = 0.10). However, no differences were found for the average number of pumps participants made for the balloons following negative feedback with small hypothetical monetary reward and that with large hypothetical monetary reward (5.98 ± 0.27 vs. 6.14 ± 0.29, *t*(19) <1, *p* = 0.60), and no differences were found for the average number of pumps participants made for the balloons following negative feedback with small hypothetical monetary reward and that with small real monetary reward (5.97 ± 0.27 vs. 6.12 ± 0.31, *t*(19) <1, *p* = 0.58).

### ERP Results

**Figure 3:**
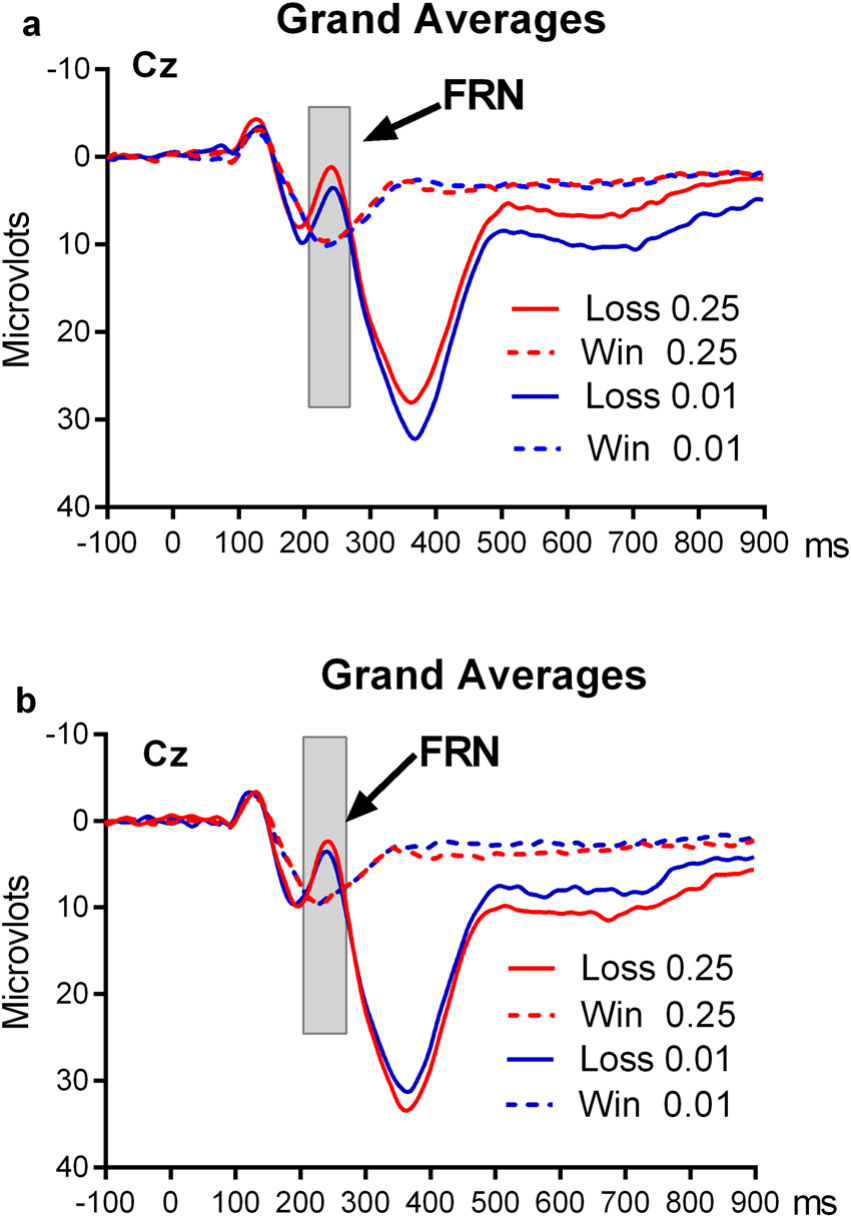
ERP waveforms. Grand average ERPs at Cz for four kinds of results between loss and win in the real money condition (**a**) and hypothetical money condition (**b**).

Consistent with previous studies^17,38–40^, we observed a robust FRN component in the central to frontal-central scalp regions. We analyzed the FRN using the mean amplitudes of ERP response to different trials in the 210–270 ms time window post-onset of feedback from the Cz location (Fig. 3). Similar to behavior data analysis, a 2 (reward condition: hypothetical vs. real) × 2 (reward magnitude: small vs. large) × 2 (feedback: positive vs. negative) repeated measures ANOVA analysis was conducted on the FRN amplitudes (Fig. 2b). This analysis revealed a significant three-way interaction among reward condition, reward magnitude, and feedback (*F* (1, 19) = 13.25, *p* = 0.02, *ω*^2^ = 0.379), and a significant main effect of feedback (*F* (1, 19) = 8.42, *p* = 0.009, *ω*^2^ = 0.274), with larger FRN (more negative-ongoing amplitude) in response to negative (loss) outcomes than positive (win) outcomes. We then separately analyzed the FRN component in response to positive and negative feedbacks using 2 (reward condition: hypothetical vs. real) × 2 (reward magnitude: small vs. large) ANOVA, respectively. For the FRN component in response to positive feedback, there were no effects of reward condition (*F* (1, 19) = 2.32, *p* = 0.144, *ω*^2^ = 0.062) or reward magnitude (*F* (1, 19) <1, *p* = 0.54), and no interaction between reward condition and reward magnitude (*F* (1, 19) <1, *p* = 0.39). For the FRN component in response to negative feedback, there was a significant interaction between reward condition and reward magnitude (*F* (1, 19) = 21.15, *p* = 0.001, *ω*^2^ = 0.505), but no effects of reward condition (*F* (1, 19) <1, *p* = 0.33) or reward magnitude (*F* (1, 19) = 1.42, *p* = 0.25). Further post-hoc comparisons indicated that the FRN in response to negative feedback with large real monetary reward was significantly more negative-ongoing than that with small real monetary reward (3.52 ± 1.29 μV vs. 5.65 ± 1.54 μV, *t*(19) = −3.69, *p* = 0.002, *ω*^2^ = 0.146), while no differences were found for the FRN in response to negative feedback with large hypothetical monetary reward compared to that with small hypothetical monetary reward (5.76 ± 1.53 μV vs. 4.81 ± 1.75 μV, *t*(19) = 1.52, *p* = 0.14).

**Figure 4:**
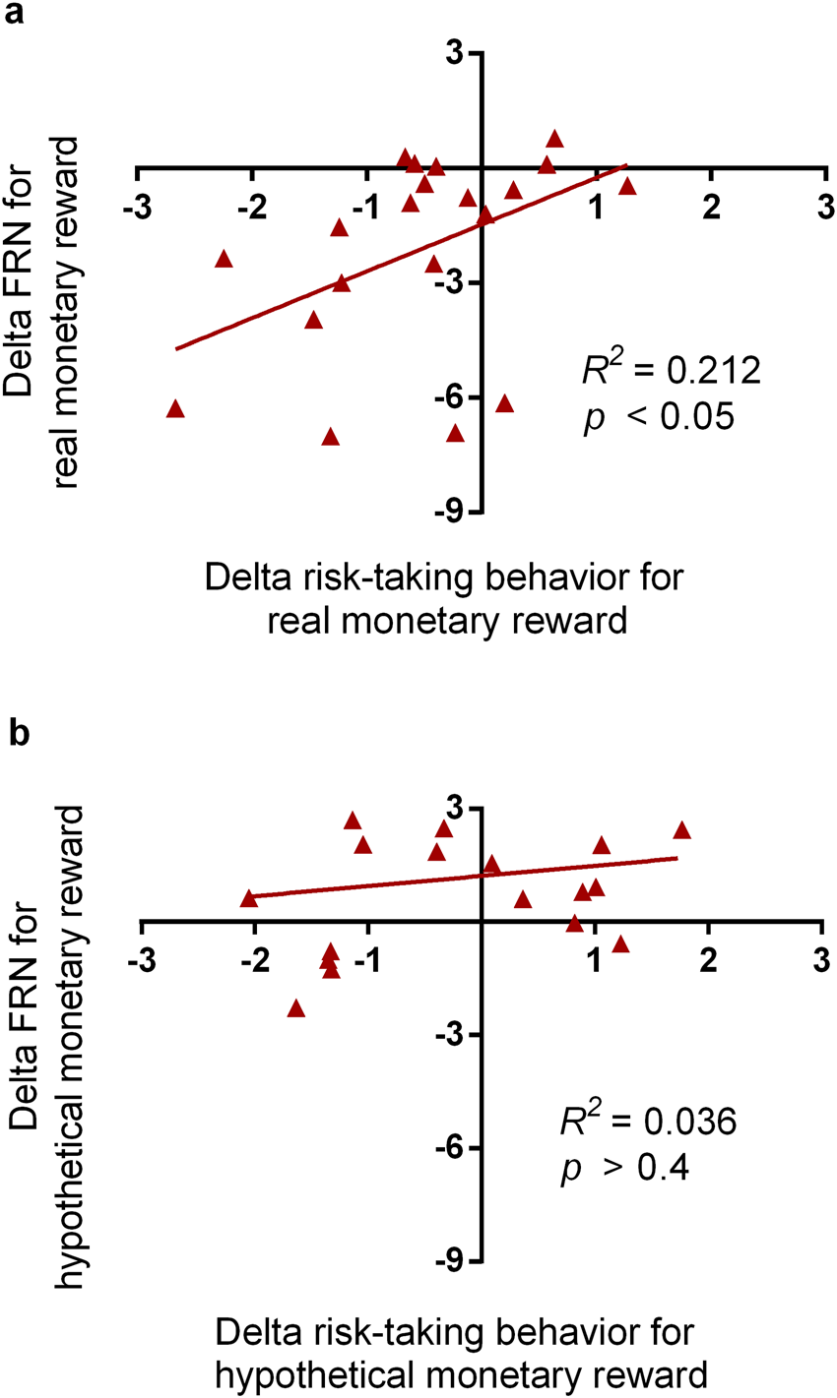
Correlations between delta FRN and delta risk-taking behavior. Delta FRN (FRN differences from small to large rewards) correlated with delta risk-taking behavior (differences in number of pumps participants made for each balloon from small to large rewards) under the real monetary reward condition (**a**) but not under the hypothetical monetary reward cikondition (**b**).

In order to examine the relationships between the FRN, risk-taking behavior, and reward conditions, a hierarchical regression analysis was conducted with the delta FRN (FRN amplitude difference between large and small rewards) as the criterion, delta risk-taking behavior (balloon inflation pump difference between large and small rewards) entered on the first step, and reward condition entered on the second step, followed by their interaction. The model accounted for 43.3% of the variance in the FRN amplitude difference (*F*(2,37) = 14.13, *p* = 0.001), with delta risk-taking behavior accounted for 10.6% of variance (*B* = 0.83, *t* = 2.13, *p* = 0.04) and reward condition accounted for 32.7% of the variance (*B* = 3.11, *t* = 4.62, *p* = 0.001), whereas the interaction did not reach statistical significance (*p* = 0.14). When correlation analyses were conducted between the delta FRN and delta risk-taking behavior in each reward condition separately, significant positive correlation was only observed in real monetary reward condition (*r* = 0.46, *p* = 0.043, Fig. 4a), but not in hypothetical monetary reward condition (*r* = 0.19, *p* = 0.44, Fig. 4b).

## Discussion

The present study examined how the magnitudes of real and hypothetical monetary reward modulate risk-taking behavior and brain activity using event-related potential with a well-validated BART paradigm. Behavioral data showed that participants made less number of balloon inflation pumps (i.e., were more risk averse) after negative feedback (loss) of large real monetary reward compared to small real monetary reward. However, there were no behavior differences between large and small hypothetical monetary rewards. These results replicated findings from a previous study showing that participants took less risk with increased magnitude of real rewards, while risk-taking behavior did not change with increased magnitude of hypothetical rewards^19^. The findings that participants were more risk averse with large real monetary reward compared to large hypothetical monetary reward are also in line with previous studies reporting less impulsive choices associated with real money^14,17–19^. However, no risk-taking behavior differences were found between small real monetary reward and small hypothetical monetary reward in this study, supporting the interactions between reward magnitude and reward authenticity on risky decision making. Taken together, these findings suggest that the magnitudes of hypothetical versus real monetary reward may modulate risk-taking and decision-making behavior in a different manner.

Similar to behavioral findings, ERP results also demonstrated differential effects of hypothetical versus real monetary reward magnitude on the FRN, a component typically elicited by unfavorable or unexpected outcome feedback^3,38,41–48^. Specifically, a significantly enlarged FRN was found in response to negative outcome (loss) during risk taking with large real monetary rewards compared to small real monetary rewards, indicating that reward magnitude of real money modulates risk taking in the brain. However, such differences were not found between the FRN components during risk taking with large hypothetical monetary rewards and small hypothetical monetary rewards, suggesting a null effect of hypothetical reward magnitude on risk taking in the brain. These findings suggest that the magnitudes of hypothetical and real monetary rewards may modulate brain responses to negative outcome during risk taking in a different manner.

The greater FRN after loss of large as opposed to small real monetary rewards is consistent with several previous studies showing significant modulation effects of reward scale or magnitude on brain activity^49^. These findings may be explained by the reward prediction error (i.e., the difference between the amount of actual reward and the expected reward) in the reinforcement learning theory^35,46,50–52^. According to this theory, the FRN reflects the activity of the anterior cingulate cortex (ACC) and codes the size of negative prediction error^45,49,53,54^. Previous human and animal neuroimaging studies have consistently demonstrated that the ACC is sensitive to reward magnitudes^54,55^. For example, a human brain imaging study investigated the neural coding of the variables contributing to the calculation of expected values and demonstrated that ACC activation reflects an integration of the outcome valence, probability and magnitude^55^. In the current study, the outcome value of large monetary rewards associated with each balloon inflation pump was 25 times larger than that of the small monetary rewards, resulting in a much larger prediction error associated with balloon explosion and money loss, therefore inducing a greater FRN component.

Another possible explanation for the observed greater FRN after loss of larger real monetary rewards is that the FRN may reflect the evaluation of emotional and motivational consequences of decision outcomes, including regret or arousal level^35,36,56–58^. Arousal level is typically increased after a loss outcome compared to a win outcome^59,60^, while regret emotion is usually induced when individuals are aware that the outcome they obtained would be better if they had made a different choice^61^. Previous studies have consistently shown that the FRN is sensitive to arousal level or emotional variables^56,62,63^, and correlated with affective ratings^37,56,64,65^. For the real monetary reward conditions, participants might have obtained higher arousal level and regret after loss of large rewards than after loss of small rewards, which elicited a stronger FRN component.

In contrast to the significant modulation effect of real monetary reward magnitude, we failed to observe a modulation effect of hypothetical monetary reward magnitude on the FRN, although the magnitudes of real and hypothetical rewards were identical. These findings are consistent with a previous study showing that the FRN was sensitive to the buying power and was influenced by true value but not the face value of the monetary rewards^66^, suggesting that the FRN is modulated by the magnitude of true value but not the hypothetical value of rewards. The FRN is believed to be generated from the ACC, which plays a key role in extracting the essential reward information in complex contexts^67,68^ and integrates various aspects of a decision including probability, payoff, and effort^69,70^. Compared to larger real monetary rewards which have greater utility for participants in the real world, increased hypothetical monetary rewards have no value in the real world. Consequently, participants’ arousal level and regret after loss of large hypothetical rewards may be similar to those after loss of small hypothetical rewards, leading to no differences in the FRN component.

According to principles of reinforcement learning, the FRN may reflect a reward prediction error signal sent from the midbrain dopamine system to the anterior cingulated cortex^45^. Given that prediction errors might signal the need to adjust behavior^71^, the FRN should reflect not only whether the current feedback is good or bad but also correlated with the future behavior. Consistent with this idea, significant correlations were found between delta FRN and delta risk-taking behavior for real monetary rewards. These findings are also in line with previous findings that the FRN following monetary loss was more negative-going when participants chose a different option on the subsequent trial^43,72–75^. However, no correlations were found between delta FRN and delta risk-taking behavior for hypothetical monetary rewards, providing further evidence supporting that real and hypothetical reward magnitudes may affect risk-taking behavior and brain activity in a different manner.

The present study has several weaknesses and limitations. First, although we used different amount of money for small versus large (1 cent vs. 25 cents per pump) reward magnitudes in the BART task, even the large rewards (25 cents per pump) were still a quite small amount of money. Nevertheless, both risk-taking behavior and the FRN component showed differential modulation effects from real versus hypothetical monetary rewards. Replication studies with a really large amount of monetary rewards are needed to validate these findings. Second, the maximal inflation capacity of each balloon trial was shortened from 128 pumps in the original BART paradigm to 12 pumps in the current modified BART paradigm due to time constraint of the study. Therefore, the BART sensitivity to measure subtle changes in individual risk-taking behavior may be reduced, which could contribute to the null findings in the overall averaged risk-taking behavior between small and hypothetical monetary rewards. Moreover, we used real losses instead of the standard “no gain” after balloon explosion in order to improve the ecological validity of risk-taking during the BART task. However, this modification may trigger loss aversion in addition to risk aversion^76^. Future studies are warranted to replicate findings from this study while maintaining the original BART parameters. Finally, the sample of this study was not large and only consisted of healthy young college students with a small age range, hence the current findings may not be generalized to other populations with a different age range and education level or clinical diseases. For example, modulation of the FRN has been suggested to be a potential biomarker in psychopathology^77^ and is impaired in schizophrenia. It will be important to examine whether the modulation effects of real versus hypothetical monetary reward magnitudes persist in these patients and further understand the FRN modulation in the reinforcement learning theory. Small samples may also bring some problems for the regression and correlation analyses between behavior and brain activity^78,79^, therefore future studies are necessary to validate the current findings with a much larger sample size.

In summary, the present study demonstrated that the magnitudes of real and hypothetical monetary rewards have differential influences on risky decision making both behaviorally and neurologically. Only the change of real monetary reward magnitude, but not the change of hypothetical monetary reward magnitude, modulates risk-taking behavior and brain activity. These findings suggest real and hypothetical money rewards may have differential validity for modulating human behavior and brain activity, therefore future studies should exercise caution when drawing conclusions on real human decisions from hypothetical studies of intended behavior, especially when using a relatively large amount of rewards.

## Methods

### Subjects

This study was approved by the Sun Yat-sen University ethics committee and was carried out in accordance with the Declaration of Helsinki. Twenty undergraduate students (12 females, mean age = 19.4 ± 1.0 years) provided written informed consent before participating in the study. All participants were right-handed and had no history of neurological or psychiatric disorders. Each participant received a basic pay of 30 Chinese Yuan (about US $4.5) to compensate for his/her participation in this study. Participants also received an additional payment (between 50–80 Chinese Yuan, about US $7.5 to $12) which was commensurate with their earnings from the real monetary reward condition.

### Tasks and Procedure

The participant sat approximately about 70 cm in front of a computer screen in an electrically shielded room and completed the BART task including 100 balloons in each scale (0.01 vs. 0.25 Chinese Yuan) condition with real or hypothetical monetary reward. For the hypothetical money condition, participants were told that the gains in the task were hypothetical monetary reward, but they would have to make decisions as though they were really going to get the rewards they chose. While for the real money condition, participants were told that the gains in the task were real and equal to the pay they got from the experiment. The order of the scale was randomized in the same type of money reward condition and the order of the money reward condition was randomized between subjects.

The BART paradigm was modified from previous studies^17,39,80^. During the task (Fig. 1), subjects were asked to inflate a balloon that could either grow larger or explode. The balloon stimuli were blue spheres with radii that increased proportionally to the amount of money added. The visual angle of the balloon before inflating is 4.5°, which increased by 0.3–0.54^o^ (depending on the amount of money added to the balloon). The delay between the subject’s response and feedback was randomized from 1 to 1.2 s. Once the balloon was pumped past its individual explosion point, a number (accumulated money for the current balloon) indicating the loss of money colored either red or green (to indicate valence of outcome) would appear. For half of the subjects, green indicated monetary gains and red indicated monetary losses, whereas the reverse mapping was used for the remaining subjects. Before the formal experiment, subjects practiced several balloons to ensure they understood the task and mastered the experimental procedure.

The task consisted of 100 trails (balloon) to increase the number of negative feedback epochs available for averaging with each reward type. The maximum breaking point for each balloon was shortened to 12 pumps to reduce the experimental time and minimize participants’ tiredness during the task. The probability of the balloon bursting after first pump was 1/11, after the second pump was 1/10, and so on, until the 12^th^ pump. However, if the balloon was pumped past its individual explosion point, participants lost all previous earnings from that balloon, and the same loss amount was subtracted from the cumulative earnings of the payment for research participation as the penalty. We used real losses after balloon explosion instead of the standard “no gain” in order to improve the ecological validity of risk-taking during the BART. This type of modification has been successfully used in our previous imaging studies of risk taking^80,81^. The experiment lasted about 20–25 min for each condition.

### EEG recording and Analysis

EEG was recorded (band-pass 0.1–100 Hz, sampling rate 512 Hz) with ANT EEG system (ANT, Enschede, Netherlands) using an elastic electrode cap with 64 Ag/AgCI electrodes in accordance with the international 10–20 system. Common average reference was used for online EEG recording and EEG signal was off-line algebraically re-referenced to the average of the left and right mastoids. Vertical and horizontal electrooculograms (EOGs) were recorded with two pairs of electrodes: one pair was placed above and below the left eye in parallel with the pupil and the other pair was placed 10 mm from the lateral canthi. Electrode impedance was maintained below 3 kΩ throughout the experiment. EEG recordings were segmented for the epoch from 100 ms before the onset of stimuli to 1000 ms after the onset, with the pre-stimulus period as the baseline. The trials contaminated by eye blinks, eye movement or muscle potentials exceeding ± 100 μv at any electrode were excluded before averaging. The data were digitally low-pass filtered below 30 Hz. ERP were then averaged for all stimuli within each session.

Based on the FRN results of the previous studies^17,38,39^ and visual inspection of our ERP waveforms, we analyzed the FRN effects using the mean amplitude of ERP response to different trials in the 210–270 ms time window from the Cz location (Fig. 3). The Greenhouse-Geisser correction for repeated measures was applied where appropriate.
